# A Volunteer Program to Connect Primary Care and the Home to Support the Health of Older Adults: A Community Case Study

**DOI:** 10.3389/fmed.2018.00048

**Published:** 2018-02-26

**Authors:** Doug Oliver, Lisa Dolovich, Larkin Lamarche, Jessica Gaber, Ernie Avilla, Mehreen Bhamani, David Price

**Affiliations:** ^1^Department of Family Medicine, McMaster University, Hamilton, ON, Canada

**Keywords:** volunteerism, primary health care, aging, interprofessional health care team, older adults

## Abstract

Primary care providers are critical in providing and optimizing health care to an aging population. This paper describes the volunteer component of a program (Health TAPESTRY) which aims to encourage the delivery of effective primary health care in novel and proactive ways. As part of the program, volunteers visited older adults in their homes and entered information regarding health risks, needs, and goals into an electronic application on a tablet computer. A total of 657 home visits were conducted by 98 volunteers, with 22.45% of volunteers completing at least 20 home visits over the course of the program. Information was summarized in a report and electronically sent to the health care team via clients’ electronic medical records. The report was reviewed by the interprofessional team who then plan ongoing care. Volunteer recruitment, screening, training, retention, and roles are described. This paper highlights the potential role of a volunteer in a unique connection between primary care providers and older adult patients in their homes.

## Introduction

Health care systems are not consistently designed to help individuals maintain or improve their health in a proactive manner. Primary care should be person-focused, community-engaged, and well-coordinated with other sectors of the health system (1, 2). Shifting the focus in primary care from reactive to proactive requires a paradigm shift in both thinking and practice, thus empowering patients and families to respond to or even prevent a crisis. This shift is even more critical as Canada’s population ages.

**Figure 1:**
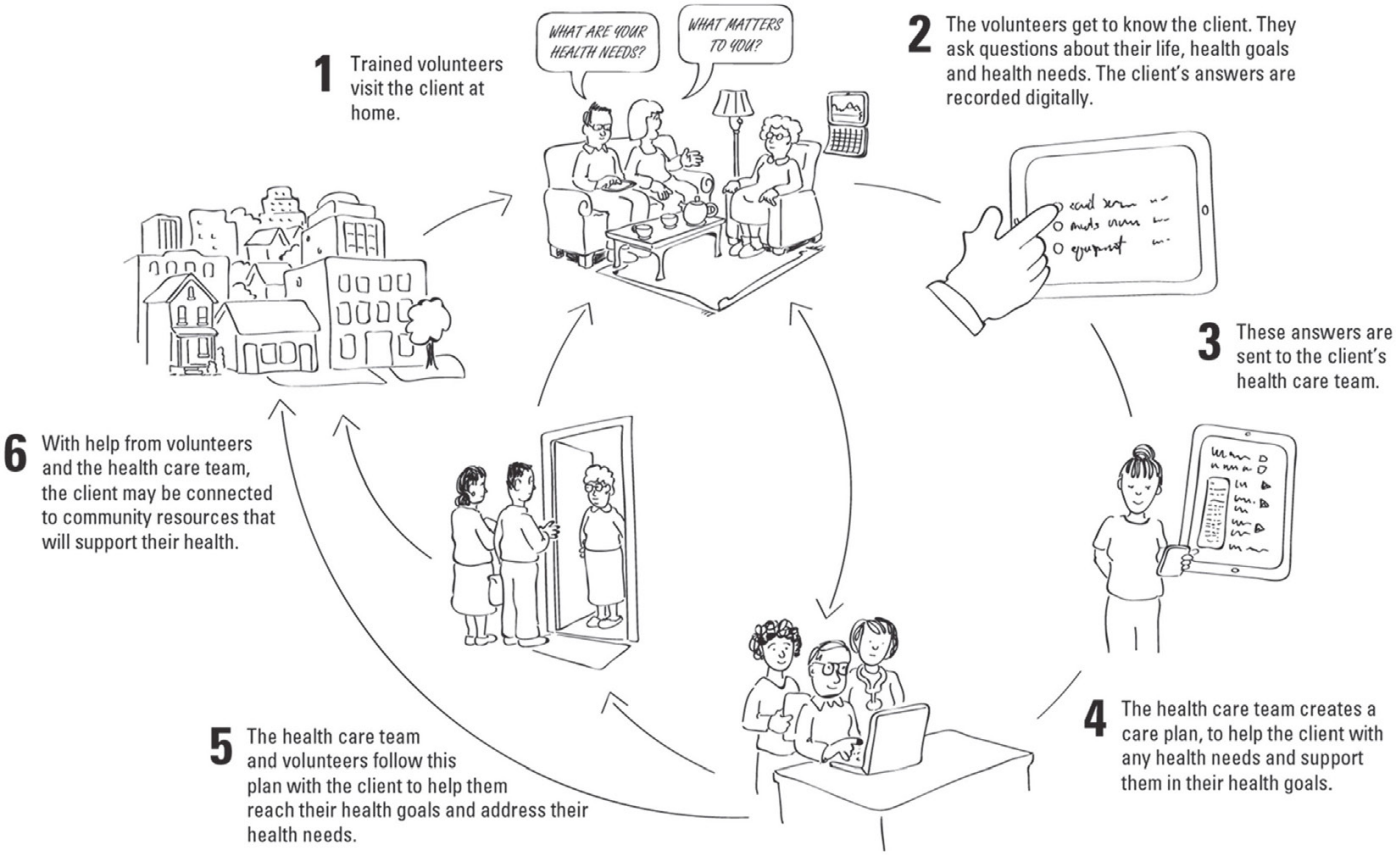
Health TAPESTRY process. Volunteers visit clients in their home and use the Health TAPESTRY Application to collect information. This information is summarized on a Health TAPESTRY-report and is uploaded into the person’s electronic medical record to be shared with the interprofessional health care team at the clinic. Reports are viewed and an action plan is developed which can include community supports, volunteer follow-up visits, and follow-up in any nature (i.e., phone call, clinic visit) by health care team, including the personal health record (kindredPHR).

The Health TAPESTRY (Health Teams Advancing Patient Experience: Strengthening Quality) approach is a model of care which aims to overcome some of these barriers in the health care system and truly shift care from reactive to proactive care. In essence, the Health TAPESTRY program promotes person-focused, proactive care that focuses on health risks, needs, and goals in an aim to help individuals experience healthier lives, for longer, in the places where they live. It aims to do this by linking four parts: trained community volunteers, interprofessional primary health care teams, innovative eHealth technologies, and community engagement. Operationally, Health TAPESTRY involves the collection of health risks, needs, and goals on the Health TAPESTRY Application (TAP-App) by volunteer pairs in the home, which are summarized in an electronic report that is uploaded into a clients’ medical record and shared with their interprofessional health care team. The health care team in our setting includes a family physician and appropriate clinicians, including an occupational therapist, physiotherapist, clinical pharmacist, dietitian, nurse practitioner, and system navigator, depending on the issues involved with clients’ care. The team engages with the client and considers their goals to create an action plan which can include community supports, volunteer follow-up visits, or follow-up by the health care team (see Figure 1). A randomized controlled trial involving 360 older adult patients aged 70 years or older has been carried out in Hamilton Ontario to gain a richer understanding of all key elements of the Health TAPESTRY program (3). The sample of patients included community-dwelling individuals rostered to the family health team who volunteered to participate in the trial. Full details of the protocol are reported elsewhere (3).

A critical part of Health TAPESTRY is its volunteer program. Research has shown that health care volunteers can play an important role in supporting patient health in communities (4, 5). There have also been benefits from the use of volunteers to organizations who use them and to the global economy overall (6, 7). However, the majority of research on the benefits of volunteers has focused on community- or hospital-based programs, demonstrating benefits from inclusion of a trained volunteer force in the delivery of support and services to patients (8, 9); the potential importance of the integration of volunteerism into primary care has not been well studied. In Health TAPESTRY, trained community volunteers work as an extension of the primary care team. Importantly, their roles are distinct but complementary to roles of paid clinical staff. The following is a community case report that describes the volunteer program developed for the trial, including the volunteer program structure role, recruitment, training, and retention.

## Methods

The objective of the volunteer program within the Health TAPESTRY program is to foster meaningful experiences for volunteers in a primary care setting. The volunteer program provides a template for fostering and integrating volunteerism into primary care settings to support health needs and goals of patients and it is described in enough detail so that communities or primary care providers interested in adopting the approach will have a template from which to work. It should be noted that ethics approval was granted (Hamilton Integrated Research Ethics Board, File #14-084).

### The Role of the Volunteer Organization

A key element of this program was ensuring that our primary health care setting (McMaster Family Health Team) was supported by a local community volunteer organization. Shalom Village is a long-term care facility in Hamilton that served as the local community volunteer organization for Heath TAPESTRY. This organization trained and assigned a volunteer coordinator who took on all tasks related to recruiting, screening, training, matching, and scheduling Health TAPESTRY volunteers.

### The Role of the Health TAPESTRY Volunteer

Volunteers were expected to form supportive relationships with clients, gather information about clients’ health needs and goals, assist clients with gaining access to their own personal health record (kindredPHR), share information about community resources, and provide overall motivational support. Table 1 includes a description of each role and the associated training involved to help accomplish these tasks.

**Table 1:** Volunteer role expectations, role descriptions, and associated training received for role expectations.

Volunteer role expectation	Role description	Training received
Form supportive relationships with clients	The longitudinal nature of the volunteer experience over a series of visits allows trust to be built between the volunteer pair and the client The clients’ knowledge that these volunteers are working as an extension of their regular primary care physician and team helps with this trust and common understanding of the purpose of the Health TAPESTRY program	Online modules focused on communication skills and cultural awareness
Gather information about client’s health needs and health-related goals	Volunteers conduct a series of validated questionnaires on topics related to healthy aging: nutrition, physical activity, polypharmacy, social connectedness, advance care planning, mental health, memory	In-person and online education sessions on use of the tablet computer, administration of the health tools, and SMART goal setting with clients
Assist clients with set-up of their own personal health record (kindredPHR)	kindredPHR allows clients to book appointments online, track their medical information, and securely message their primary care team Volunteers are trained to use the PHR and assist clients with password selection, log-on, and authentication	Online and in-person learning about kindredPHR Ongoing support from Department of Family Medicine IT team
Share information about community resources	Volunteers can receive information from clinics that they can share in person with clients as part of the ongoing care plan Volunteers are assisting with the piloting of the EU-GENIE navigation tool that helps to customize online searches for clients’ needs/location	In-person training on use of community program databases (inform Hamilton; Ontario211; EU-GENIE)
Provide motivational support	Can support and motivate clients in achieving their self-designed health goals	In-person role play sessions and training on SMART goals

In addition to roles that volunteers had with clients, defined in Table 1, volunteers also had additional tasks to complete before, during, and after each client visit. Volunteers were asked to complete a detailed activity log and a narrative reflection about their own individual experience with the client. At the beginning of their participation in the program, volunteers were asked to create clear objectives for their own participation. These tasks were helpful to understand volunteers’ experiences in the program.

### Volunteer Recruitment

The volunteer coordinator used several strategies to recruit volunteers for the program. Email and printed brochures were distributed to organizations in the community and on the McMaster University campus (e.g., Victorian Order of Nurses, McMaster Student Success Centre, and Health Sciences undergraduate classes). Advertisements also ran in local newspapers and online through a local volunteer database containing volunteer opportunities in the region. Word-of-mouth was also an important form of recruitment as active volunteers began speaking about their experience to friends in the community (see Table 2 for proportion by recruitment source).

**Table 2:** Recruitment sources.

Recruitment source	*N*(proportion, %)
Word-of-mouth	25 (17.5)
Newspaper	24 (16.8)
Posting/flyer	26 (18.2)
McMaster University Student Centre	30 (21.0)
McMaster website	4 (2.8)
Other	11 (7.7)
Unknown	23 (16.1)

### Volunteer Screening

Before training, applicants, who were required to be aged 18 or older, were asked to complete the written application, participate in an in-person interview with the volunteer coordinator and provide two character references, a police vulnerable sector check and proof of a TB skin test. The volunteer coordinator managed all applications and scheduled all in-person interviews.

### Volunteer Training Program

Volunteers who passed the screening process were then required to complete the Health TAPESTRY volunteer training program which used a blended-learning approach including in-person and online components. The curriculum was created using a combination of local expert opinion and review of the current literature (10). The in-person training involved a 2-h interactive session that provided an overview of Health TAPESTRY and the volunteer role, more detailed information about client privacy and confidentiality as well as volunteer health and safety. This session also provided the volunteers practice with the technology and role play exercises.

The online component involved an interactive online training center, the Health TAPESTRY Virtual Learning Centre (VLC). This was created to allow volunteers to go through the core skills and topics in their own time and at their own pace. Learning modules on the VLC included live action scenarios, PowerPoint presentations with voice-overs, and testimonials from Health TAPESTRY volunteers. Time spent on each learning module and scores on content quizzes were tracked (see Table 3). This included in-person and online training sessions as well as ongoing learning as described below. The average time spent in training was 11.5 h. Successful applicants who completed their training were required to sign a confidentiality agreement and waiver prior to being scheduled for any home visits.

**Table 3:** Modules and other features on the Virtual Learning Centre (VLC).

Module name	Chapters/Quiz
Introduction	Greeting from the Volunteer coordinator
	A Word from Other Volunteers

Effective Communication	Effective Communication
	Tips for Communicating with Older Adults
	Communicating with Older Adults: Case Studies
	Effective Communication Quiz (14 questions)

Conflict Resolution	Conflict Resolution
	Conflict Resolution Quiz (9 questions)

Program Implementation	Program Implementation
	Program Implementation Quiz (13 questions)

Intercultural Communications	Intercultural Communications
	Intercultural Communication Sensitivity
	Intercultural Communication Quiz (14 questions)

Data Gathering Tools	Duke Social Support Scale
	Edmonton Frail Scale
	EQ-5D—Quality of Life
	Screen II—Nutrition

Privacy and Confidentiality	Privacy and Confidentiality
	Privacy and Confidentiality (13 questions)

Health and Safety	Personal Safety
	Infection and Prevention
	Health and Safety Quiz (13 questions)

Information Technology	How to use an iPad
	The TAP-App
	Information Technology Quiz (14 questions)

McMaster PHR	PHR
	McMaster PHR (now kindredPHR)

Mental Health	Recognizing Clients in Need of Help during a Volunteer Visit
	Mental Illness in Older Adults
	Mental Health Quiz (10 questions)

**Other features of VLC**	**Description**

Discussion board	To allow Health TAPESTRY volunteers and volunteer coordinator to share experiences and learnings in the program

Dashboard	To track progress on modules

Entry/exit surveys	Assess various aspects regarding the volunteers

#### Ongoing Learning (Retention Methods)

In addition to the VLC and in-person sessions described above, further in-person sessions were delivered by health care experts or program staff to foster retention and ongoing learning for volunteers in the program. Examples included Bone Health & Fall Prevention, Advance Care Planning, Common Infections in Community Dwelling Older Adults, and Goal Attainment. Each session was evaluated with a brief evaluation form.

### Volunteer Management System

The volunteer coordinator used an administrative database (Volunteer Management System) on the TAP-App to manage volunteer schedules and demographic information related to volunteers. In addition, this database allowed tracking of client home visits, submission of reports by volunteers and completion of narrative reflections and visit notes associated with home visits. Volunteers visited the homes of clients in pairs. The pairs were matched based on differences in experience with volunteering in a health care role. For example, a more seasoned volunteer who reported feeling relatively comfortable in their volunteer role (e.g., retired teacher and retired nurse) was matched with a more novice volunteer who lacked experience working with older adults or in a home-based setting (e.g., undergraduate student).

## Results

In total, 143 volunteers were recruited and applied to volunteer. The success of the recruitment strategies was relatively uniformly distributed across four sources, with the university student center resulting in the highest recruitment rate (see Table 2). A total of 98 volunteers saw at least one client, of these, 22 (22.45%) were considered highly engaged throughout the duration of implementation (attended 20 or more client home visits). It should be noted that there were changes in volunteer status (*n* = 45), such that volunteers were no longer interested or unable to contribute to the program (i.e., moved, too busy, personal reasons, did not complete training).

### Volunteers in the Home

In total, 657 home visits were conducted. Volunteers were sent on home visits in pairs to ensure optimal safety and efficient completion of assigned tasks. The frequency and number of home visits to a particular client depended on the wishes of the client and on how much time was required for the volunteer pair to complete all relevant surveys. The average duration to collect all client information about health goals and needs on the TAP-App was 1 h 20 min (excluding travel), in addition to unrecorded interaction time during which volunteers were building rapport before or after the TAP-App was administered (i.e., chit chat, client story telling). The typical number of home visits needed to compete the information on the TAP-App was 3–4 home visits, including a scheduled 3-month follow-up visit after all initial information had been collected and sent to the primary care team.

### Retention Methods

A total of eight ongoing learning sessions were held on topics of specific need or interest to volunteers during the course of the project, such as mental health in the aging population, helping clients with advance care planning, and fitness, osteoarthritis, and falls prevention. On average, 20 volunteers attended each session. In general, the sessions were rated positively in terms of providing new and relevant information, and providing the information in an engaging and meaningful way.

### Volunteer Program Costs

The costs of running a volunteer program such as Health TAPESTRY vary depending on the number of clients needed. Based on our experience, a good ratio of clients to volunteers is 3:1. Using the example of 120 clients and 40 volunteers, the estimated cost of running this volunteer program is $22,650 per year (see Table 4 for a cost breakdown).

**Table 4:** One-year cost of a volunteer program (based on 120 clients and 40 volunteers).

Item	Item cost	Total Cost
Volunteer recruitment (promotion, advertising)	–	$1000
Volunteer coordinator—salary and benefits	$25.69/h	$17,263.68^a^
Volunteer police checks^b^	$0 each	$0
Volunteer TB tests^d^	$0 each	$0
Volunteer training (catering, parking costs)^c^	$20 each	$800
Volunteer refresher (catering, parking costs)^c^	$20 each	$800
Virtual Learning Centre use	$350 set-up, $120 monthly	$1,790
Office (printing, faxing, supplies)	–	$1,000

TOTAL	–	$22,653.68

*^a^Based on two 7 h days/week*.

*^b^Free if requested online*.

*^c^Assumes room rental is in-house (free of charge) and already includes WiFi*.

*^d^Covered by provincial healthcare, administered by family doctor*.

## Discussion

The Health TAPESTRY volunteer program is unique in that it draws in energetic and skilled volunteers who are looking to make a difference in the lives of older adults and allows them to serve as a valuable connector to the patient’s primary care team. To our knowledge, there is nothing similar to it in primary care. Strengths of the program revolve around providing meaningful experiences for volunteers in supporting clients’ health needs and goals. By serving as an extra set of eyes and ears in the homes of patients, Health TAPESTRY volunteers play a key role in supporting the primary care team. The integration of a clinician champion within the volunteer program was a key element to success as this element ensured that clinicians had a say in the development and implementation of the volunteer program. This integration also meant that more efficient and immediate course-corrections could be made in training and implementation where required. It is recommended for any volunteer program in primary care of this nature to have a clinician champion as an essential element. Another strength of the volunteer program was the flexibility built into the training model itself. For example, mental health emerged as a critical issue related to home visits. After identifying this learning gap for our volunteers, a training module to address these concerns was created. This new material was delivered as a live presentation for active volunteers and then deployed to the VLC for present and future volunteers to complete the module. Thus, it is recommended that the training program be flexible and adaptive to changing training needs as the program evolves.

The volunteer program did not come without its challenges. Managing the competing schedules of volunteer pairs was a time consuming exercise and required frequent updating, particularly with the use of university students in our volunteer pool. Recruitment of these enthusiastic volunteers was not a challenge, but retention and scheduling took ongoing time and effort due to their busy and unpredictable schedules. We suggest providing meaningful ongoing training as a retention strategy to address changing needs of the volunteers and maintain meaningful contact “on the ground.” We found the lunch and learn format particularly helpful for ongoing training opportunities and as a way for volunteers to directly connect with the clinician champion. Another challenge for some volunteers was transportation. In particular, many student volunteers did not have a vehicle, so getting to and from client homes took extra planning. In the end, this became another opportunity for many volunteer pairs to bond, as experienced volunteers would often pick up the student volunteer to go to the client’s home together.

Finally, the financial costs of running a volunteer program need to be considered and balanced against potential cost-savings involved in shifting care away from acute care hospitals to a prevention-based primary care setting. Although there have been demonstrated cost-savings using volunteers in health care (11), volunteers require adequate training on key program areas including client privacy and confidentiality, health and safety, and any responsibilities they are tasked with. Primary care teams and community organizations interested in implementing a volunteer program connected to primary care need to consider the costs of $22,650 per year to ensure the program is resources for effective delivery. The majority of this cost is associated with a paid volunteer coordinator. A published evaluation of the effectiveness of the program on client outcomes, as well as an exploration of the volunteer experience, will be forth coming.

### Conclusion

Recruiting, training, and integrating trained community volunteers into a primary care setting are possible. The Health TAPESTRY volunteer program provides a rewarding experience for volunteers, through meaningful contributions to the care of older adults in a primary care setting. It is a unique program that weaves together and connects community resources and organizations, the interprofessional primary care team support, and volunteers to the client in their home to support their health risk, needs, and goals.

## Ethics Statement

Ethics approval was granted (Hamilton Integrated Research Ethics Board, File #14-084).

## Author Contributions

All authors contributed to the conception of the idea of the Health TAPESTRY volunteer program volunteer program and the preparation of this manuscript. DP, LD, and DO held joint responsibility for administrative and management oversight of the Health TAPESTRY program. DP and LD wrote the initial funding proposal and overall protocol. LD, DO, and DP provided expertise surrounding aspects of primary health care and healthy aging of older adults and chronic disease management. DO and EA provided leadership in the development of the volunteer program. DO, LD, LL, JG, EA, and MB helped develop the scientific and operational aspects of volunteer program.

## Conflict of Interest Statement

We declare that the research was conducted in the absence of any commercial or financial relationships that could be construed as a potential conflict of interest.
